# Management of Mango Hopper, *Idioscopus clypealis,* Using Chemical Insecticides and Neem Oil

**DOI:** 10.1155/2014/709614

**Published:** 2014-07-22

**Authors:** S. M. Adnan, M. M. Uddin, M. J. Alam, M. S. Islam, M. A. Kashem, M. Y. Rafii, M. A. Latif

**Affiliations:** ^1^Bangladesh Agricultural University, Mymensingh 2202, Bangladesh; ^2^Bangladesh Institute of Nuclear Agriculture, Mymensingh 2202, Bangladesh; ^3^Institute of Tropical Agriculture (ITA), University Putra Malaysia, 43400 Serdang, Selangor, Malaysia; ^4^Department of Crop Science, Faculty of Agriculture, University Putra Malaysia, 43400 Serdang, Selangor, Malaysia; ^5^Bangladesh Rice Research Institute, Gazipur 1701, Bangladesh

## Abstract

An experiment was conducted in Field Laboratory, Department of Entomology at Bangladesh Agricultural University, Mymensingh, during 2013 to manage the mango hopper, *Idioscopus clypealis* L, using three chemical insecticides, Imidacloprid (0.3%), Endosulfan (0.5%), and Cypermethrin (0.4%), and natural Neem oil (3%) with three replications of each. All the treatments were significantly effective in managing mango hopper in comparison to the control. Imidacloprid showed the highest efficacy in percentage of reduction of hopper population (92.50 ± 9.02) at 72 hours after treatment in case of 2nd spray. It also showed the highest overall percentage of reduction (88.59 ± 8.64) of hopper population and less toxicity to natural enemies including green ant, spider, and lacewing of mango hopper. In case of biopesticide, azadirachtin based Neem oil was found effective against mango hopper as 48.35, 60.15, and 56.54% reduction after 24, 72, and 168 hours of spraying, respectively, which was comparable with Cypermethrin as there was no statistically significant difference after 168 hours of spray. Natural enemies were also higher after 1st and 2nd spray in case of Neem oil.

## 1. Introduction

Mango (*Mangifera indica* Linn.) is a very important and popular fruit in the world. It is the choicest fruit of the subcontinent and is known as king of all fruits. Its popularity is mainly due to its excellent flavour, delicious taste, and high nutritive value being rich in vitamins A and C. Its origin is believed to be south Asia where it has been cultivated for the last four thousand years [1]. Now it is a commercially cultivated important fruit of this subtropical region particularly Bangladesh, India, and Pakistan. But production of mango is enormously handicapped by the ravages of insect pests from seedling to their maturity. More than 300 insect pests have been recorded to attack mango crop in different regions of world [2]. Among the mango pests, Mango hopper* Idioscopus clypealis* (Lethierry) is one of the most serious and widespread pests throughout the country, which causes heavy damage to mango crop. Both the nymphs and adults of the hoppers puncture and suck the sap from tender shoots, inflorescences, and leaves of mango crop, which cause nonsetting of flowers and dropping of immature fruits, thereby reducing the yield. Hoppers also excrete a secretion, called honey dew. In moist weather, it encourages the development of fungi like* Meliola mangiferae* (Earle), resulting in growth of sooty mould on dorsal surface of leaves, branches, and fruits. This black coating interferes with the normal photosynthetic activity of the plant, ultimately resulting in nonsetting of flowers and dropping of immature fruits. This damage is called honey dew disease. On heavily infested trees, crop losses of 50% or more have been recorded [2]. In the past its control was based purely on chemicals especially synthetic insecticides. But nonjudicious application of highly toxic and persistent insecticides is causing several problems such as disrupting natural enemy complexes, development of insecticide resistance, secondary pest outbreak, pest resurgence, and environmental pollution [3]. To solve these problems farmers can shift from the unilateral reliance on insecticide use to alternative approaches. In this situation, biodegradable substitutes are now being strongly conceived by all scientists of the world. Biologically active natural plant products may play a significant role in this regard as they are environmentally safe, biodegradable, and cost effective. A large number of investigators isolated and identified several chemical compounds from leaves and seeds of many plant species and screened out many insect feeding deterrents and growth inhibitors [4]. Among them Neem based products have extensively been used and have proved their pest control efficacy against several insect pests both in field and storage. However, exploration on the use of botanicals against mango pests is scanty in Bangladesh. Under this circumstance, the present research was undertaken to manage mango hopper,* I. clypealis* (Lethierry), using three nonpersistence chemical insecticides, namely, Imidacloprid, Endosulfan, Cypermethrin, and natural product Neem oil as well as to assess their toxic effects on natural enemies of mango hopper.

## 2. Materials and Methods

The experiment was conducted in the Entomology Field Laboratory at Bangladesh Agricultural University, Mymensingh. The experiment was performed following randomized complete block design (RCBD) having five treatments including control with three replications of each treatment. Five inflorescences from five different branches of the same tree were selected alternatively from top, middle, and bottom and were sprayed with each treatment. Selection of inflorescence was done as modified method developed by [5]. The treatments were three insecticides, namely, Imidacloprid, Endosulfan, Cypermethrin, and natural Neem oil. A control treatment was always maintained with three replications. Insecticides were sprayed as recommended dose and Neem oil was used as 3% concentration (Table 1). Application of treatments and collection of data were done before 10 a.m. Data were counted on the number of hopper per inflorescence. Pretreatment data were taken just before and posttreatment data were recorded after 24, 72, and 168 hours of spraying. The percentage reduction of insect population was computed using Henderson-Tilton's formula, that is, %efficacy = [1−Ta/Ca×Cb/Tb] × 100 [6], where, Tb is the infestation in the treated plot before treatment, Ta is the infestation in the treated plot after treatment, Cb is the infestation in the control plot before treatment, and Ca is the infestation in the control plot after treatment.

Data were analyzed following ANOVA using statistical package SPSS (version 14.0). Significant differences among the means of different treatments were tested using Duncan multiple range test (DMRT). Efficacy of insecticides and Neem oil were tested on the basis of percentage reduction of hopper population. Along with this, effects of selected insecticides and Neem oil on natural enemies were assessed by taking pretreatment data just before spray and posttreatment data at 24 HAT of 1st, 2nd, and 3rd spray.

## 3. Results and Discussion

### 3.1. Effect of Three Synthetic Insecticides in Controlling Mango Hopper

Reduction in population indicated that all the tested insecticides were effective against mango hopper (Table 2). The percentage reduction over pretreatment in comparison with control plot was higher with Imidacloprid and Endosulfan followed by Cypermethrin and Neem oil. There was significant difference (*P* < 0.05) among the treatments as overall efficacy. In case of conventional insecticides, Imidacloprid was found highly effective with 83.63% reduction after 24 hours of first spray with increasing trend in efficacy as 89.97% after 72 hours; however its effectiveness decreased at 168 hours to 85%, whereas Endosulfan proved to be a good controlling agent with 75.79% reduction at 24 hours with decreasing trend after 72 and 168 hours of spray with 72.48 and 67.47% reduction, respectively. Cypermethrin was comparatively less effective than Imidacloprid and Endosulfan as it gave 64.40% reduction after 24 hours though it increased after 72 hours to 68.23% reduction but again after 168 hours of first spray 57.95% reduction was observed. Similar results were reported by [7] with significant dominance of Imidacloprid among different pesticides against okra jassid (*Amrasca biguttula biguttula*) [8]. The performance of different doses of Imidacloprid as an agent of seed and root treatment was assessed and found effective up to 45 days after treatment against chilli thrips. It was found that Imidacloprid is the most effective against mustard aphid compared to Endosulfan and Neem oil [9], whereas [10] found Endosulfan very effective against jassids on okra crop. The effectiveness of Endosulfan and Cypermethrin against aphid on okra and brinjal crops at different time intervals was evaluated and it was found that Endosulfan is more effective than Cypermethrin on brinjal crop and vice versa in case of okra crop [11, 12].

The effect of Endosulfan and azadirachtin was studied and azadirachtin was found to be moderately effective against brinjal shoot and fruit borer when used alone, whereas it varied in efficacy when used in combination such as Endosulfan + Bt (*Bacillus thuringiensis*) and azadirachtin + Bt [13]. However, in any case azadirachtin was found comparatively less effective than Endosulfan. Whereas similar combinations of pesticides along with Cypermethrin against aphids and jassids of okra and observed significant dominance of some pesticides among others, Cypermethrin showed moderate but yet more effective than Neem product (azadirachtin).

### 3.2. Effect of Natural Neem Oil in Controlling Mango Hopper

Unlike synthetic pesticides plant based pesticides have diverse pest control properties. Plant products affect different physiological processes in insects like metamorphosis including insect growth regulation, adult fertility, and toxicity and also have antifeedant and oviposition deterrent effects [14].

It was reported that they are environmental friendly; therefore, they seem to have some superiority over synthetic pesticides [15]. Moreover, a variety of plant species are available with diverse types of controlling effects as over 2400 plants have been identified with pest control properties in this respect [16].

In case of biopesticides, azadirachtin based Neem oil was found effective against Mango hopper at the rate of 48.35, 60.15, and 56.54% reduction after 24, 72, and 168 hours, respectively, which was comparable with Cypermethrin as there was no statistically significant difference after 168 hours of spray (*P* < 0.05) (Table 2). The efficacy of different Neem based readymade products was evaluated and observed to be comparatively less effective against sucking pests [17] than Endosulfan but proved superior to untreated plot. The work also supports our findings [18] and the authors tested the field efficacy of azadirachtin-A, its stable derivative tetrahydroazadirachtin-A (THA), and NeemAzal (NZ) pesticides, in comparison with Endosulfan against the complex pests of okra including jassid and whitefly, and found azadirachtin-A effective for up to 7 days whereas THA had potentiality to control the pests for up to 10 days.

Endosulfan was found to be most effective with 82.9% reduction in whitefly population followed by THA, Aza-A, and NZ (60%, 58.7% and 57.5%). Against jassid, it was reduced by 62% with Endosulfan followed by THA, Aza-A, and NZ as 40.2, 35.1, and 31%, respectively. The effectiveness of Biosal (Neem formulation) in comparison with Endosulfan and Profenofos against jassid on brinjal at different time intervals was evaluated and a moderate effect of Biosal against jassid was found with 47% mortality [19]. Crude extracts of Neem and Margosan-O (pesticide of Neem origin from USA, containing 0.3% azadirachtin as active ingredient) were tested in comparison with Malathion 57 EC [20] against white fly on brinjal and effectiveness was found as Malathion > Margosan > crude Neem extracts after 48 hours, while after 96 hours crude Neem extracts were more persistent than Margosan and Malathion, respectively. Similarly different attempts were made to test the efficacy of Neem preparations against aphid and whitefly [21, 22]. Neem extract was found effective but inferior to Imidacloprid against the spread of okra yellow vein mosaic virus by controlling whitefly population [23], whereas Neem extract was found more effective against jassid, white fly, and thrips on cotton as compared to Perfekthion which lost its efficacy after 4 days, while Neem product was persistent for up to 6 days and was much safer and nonpolluting. In our present study, Neem oil was also effective for up to 7 days [23]. Reference [24] reported that significant reduction in the population of jassid, whitefly, and thrips on cotton was up to 168 hours when Neem oil was used as 2% and Neem seed water extract as 3% but efficacy declined at 336 hours. Neem oil was effective with 56, 54, and 57% reduction in the population of jassid, whitefly, and thrips, respectively, while Neem seed water extract was relatively less effective with 49, 46, and 54% reduction against three insects. Imidacloprid, Endosulfan, Cypermethrin, and Neem oil showed the average reduction (overall performance) of hopper population in three sprays as 88.59, 73.32, 64.87, and 55.91%, respectively.

There was a significant variation among the treatments considering the number of sprays but they were statistically identical in respect of time after spray (Table 3).

### 3.3. Effect of Three Chemical Insecticides and Neem Oil on Natural Enemies

Effect of different insecticides and Neem oil on natural enemies of mango hopper, namely, green ant, spider, and lacewing varied significantly (Table 4). Among the four treatments Neem oil showed least toxicity on all the natural enemies (green ant, spider, and lacewing). Imidacloprid and Cypermethrin were moderately toxic to the natural enemies of mango hopper, whereas Endosulfan reduced hopper population significantly but it was highly toxic to the natural enemies of mango hopper (Table 4). The highest number of natural enemies' population was always observed in control plot.

This finding was in agreement with [25] who reported that Endosulfan was highly toxic to the predators of potato leaf hopper. It could be concluded from the findings of the present study that sole dependency on conventional insecticides may easily be modified by incorporating Neem oil in an environment friendly management program for mango hopper.

## 4. Conclusion

Chemical insecticides, Imidacloprid (0.3%), Endosulfan (0.5%), Cypermethrin (0.4%), and Neem oil (3%) were effective in managing mango hopper in comparison to the control. Imidacloprid showed the highest efficacy in percentage of reduction of hopper population at 72 hours after treatment in case of 2nd spray. It also showed the highest overall percentage of reduction of hopper population and less toxicity to natural enemies of mango hopper. In case of biopesticide, azadirachtin based Neem oil was found effective against Mango hopper as 48.35, 60.15, and 56.54% reduction after 24, 72, and 168 hours, respectively. The population of natural enemies was also found higher in case of Neem oil even after 1st and 2nd spray. So, it could be concluded that sole dependency on conventional insecticides may easily be modified by incorporating Neem oil as an environment friendly management program for mango hopper.

## Figures and Tables

**Table 1:** Insecticides used in efficacy trials against mango hopper.

Pesticides		Type	Source	Dose
Common name	Trade name			
Imidacloprid	Imidacloprid 25 WP	Neonicotinoid	Arysta LifeScience	0.3%

Endosulfan	Thiodan 35 EC	Organochlorine	Bayer CropScience	0.5%

Cypermethrin	Cypermethrin 20 EC	Pyrethroid	Syngenta	0.4%

Neem oil		Botanical	Laboratory of Bangladesh Agricultural University (BAU)	3%

**Table 2:** Percentage of efficacy of three insecticides against mango hopper.

Treatment	Percentage of population reduction			
	24 h	72 h	168 h	Mean
1st spray				
Imidacloprid	83.63 ± 8.91^a^	89.97 ± 10.06^a^	85.05 ± 10.94^a^	86.22 ± 9.13^a^
Endosulfan	75.79 ± 9.06^ab^	72.48 ± 9.42^ab^	67.47 ± 11.71^ab^	71.91 ± 9.49^b^
Cypermethrin	64.40 ± 10.98^b^	68.23 ± 11.72^b^	57.95 ± 10.20^b^	63.53 ± 10.53^bc^
Neem oil	48.35 ± 7.91^c^	60.15 ± 10.84^b^	56.54 ± 10.65^b^	55.01 ± 9.89^c^
2nd spray				
Imidacloprid	88.79 ± 1015^a^	92.50 ± 9.02^a^	90.12 ± 9.85^a^	90.47 ± 8.54^a^
Endosulfan	80.61 ± 10.48^ab^	72.48 ± 9.42^ab^	69.25 ± 9.11^b^	75.04 ± 9.84^b^
Cypermethrin	66.77 ± 9.26^bc^	70.85 ± 9.81^bc^	66.30 ± 9.13^b^	67.97 ± 8.43^b^
Neem oil	49.54 ± 9.42^c^	58.88 ± 11.81^c^	57.07 ± 11.93^b^	55.16 ± 10.54^c^
3rd spray				
Imidacloprid	88.42 ± 10.04^a^	90.18 ± 9.87^a^	88.65 ± 1.06^a^	89.08 ± 8.69^a^
Endosulfan	75.62 ± 11.17^ab^	72.97 ± 10.08^ab^	70.44 ± 11.84^b^	73.01 ± 9.83^b^
Cypermethrin	62.59 ± 11.89^bc^	66.45 ± 9.19^b^	60.25 ± 9.20^b^	63.10 ± 9.22^bc^
Neem oil	52.31 ± 10.30^c^	62.12 ± 11.81^b^	58.23 ± 12.90^b^	57.55 ± 10.41^c^
Overall percentage of efficacy				
Imidacloprid	88.59 ± 8.64^a^			
Endosulfan	73.32 ± 9.43^b^			
Cypermethrin	64.87 ± 9.33^c^			
Neem oil	55.91 ± 9.95^d^			

Values sharing the same letter(s) in a column are not significantly different at *P* = 0.05.

**Table 3:** Efficacy of three insecticides and Neem oil: effect of spray and time.

Treatment	Percentage of population reduction			
	Imidacloprid	Endosulfan	Cypermethrin	Neem oil
Spray				
Spray 1	86.22 ± 9.13^a^	71.91 ± 9.49^b^	63.53 ± 10.53^bc^	55.01 ± 9.89^c^
Spray 2	90.47 ± 8.54^a^	75.04 ± 9.84^b^	67.97 ± 8.43^b^	55.16 ± 10.54^c^
Spray 3	89.08 ± 8.69^a^	73.01 ± 9.83^b^	63.10 ± 9.22^bc^	57.55 ± 10.41^c^
Time (h)				
24 h	86.95 ± 8.77^a^	77.34 ± 9.23^a^	64.59 ± 9.50^a^	50.07 ± 8.12^a^
72 h	90.88 ± 8.45^a^	73.57 ± 8.58^a^	68.57 ± 9.12^a^	60.38 ± 9.39^a^
168 h	87.94 ± 9.20^a^	69.06 ± 9.58^a^	61.50 ± 9.05^a^	57.28 ± 10.15^a^

Values sharing the same letter(s) in a column are not significantly different at *P* = 0.05.

**Table 4:** Effect of three chemical insecticides and Neem oil on natural enemies.

Treatments	Number of green ants				Number of spiders				Number of lacewings			
	Before spray	At 1st spray	At 2nd spray	At 3rd spray	Before spray	At 1st spray	At 2nd spray	At 3rd spray	Before spray	At 1st spray	At 2nd spray	At 3rd spray
Imidacloprid	4.33	2.95^bc^	3.67^b^	3.82^b^	3.23	2.87^b^	3.45^b^	2.31^b^	0.87	0.74^b^	0.56^b^	0.83^b^
Endosulfan	3.98	1.14^c^	1.01^c^	1.21^c^	4.78	0.92^c^	0.87^c^	0.68^c^	0.65	0.13^c^	0.06^c^	0.19^c^
Cypermethrin	4.12	2.65^bc^	3.34^b^	5.54^ab^	5.98	2.12^b^	1.98^b^	2.43^b^	1.87	1.62^b^	1.74^b^	1.41^b^
Neem oil	4.89	3.18^b^	4.11^b^	3.65^b^	3.75	4.34^b^	2.76^b^	2.13^b^	1.43	0.98^b^	1.30^b^	0.98^b^
Control	4.98	7.65^a^	6.98^a^	7.76^a^	3.62	5.43^a^	7.98^a^	4.61^a^	1.32	2.97^a^	2.65^a^	2.13^a^
Level of significance	∗∗				∗∗				∗∗			

Values sharing the same letter(s) in a column are not significantly different at *P* = 0.05.
